# Moral growth mindset is associated with change in voluntary service engagement

**DOI:** 10.1371/journal.pone.0202327

**Published:** 2018-08-15

**Authors:** Hyemin Han, Youn-Jeng Choi, Kelsie J. Dawson, Changwoo Jeong

**Affiliations:** 1 Educational Psychology Program, University of Alabama, Tuscaloosa, AL, United States of America; 2 Educational Research Program, University of Alabama, Tuscaloosa, AL, United States of America; 3 Changwoo Jeong, Department of Ethics Education, Seoul National University, Seoul, South Korea; University of Vienna, AUSTRIA

## Abstract

Incremental implicit theories are associated with a belief regarding it is possible to improve one’s intelligence or ability through efforts. Previous studies have demonstrated that incremental implicit theories contributed to better academic achievement and positive youth development. Our study aimed to examine whether incremental implicit theories of morality significantly influenced change in students’ engagement in voluntary service activities. In our study, 54 Korean college students for Study 1 and 180 Korean 8^th^ graders for Study 2 were recruited to conduct two two-wave studies. We surveyed participants’ implicit theories of morality and participation in voluntary service activities. The effect of implicit theories of morality on change in service engagement was analyzed through regression analysis. In Study 1, the moral growth mindset significantly moderated longitudinal change in service engagement. In Study 2, the moral growth mindset significantly influenced engagement in art-related activities, while it significantly moderated change in engagement in youth-related activities.

## Introduction

Intention to engage in prosocial behavior, such as various civic activities, is a foundational source producing actual prosocial behavioral outcomes. Previous longitudinal studies have demonstrated that the presence of prosocial intention is closely associated with actual prosocial engagement [1–3]. However, the mere presence of prosocial intention does not necessarily produce prosocial behavioral outcome in the reality. Exemplar studies have reported that moral and civic exemplars who committed to moral and prosocial ends for the long term showed long-term intention as well as actual action plans and behavioral engagement [4–6]. Given these results, both intention and actual behavioral engagement are fundamental to shape persistent motivation for prosocial commitment.

However, there have been few previous studies that have examined whether characteristic traits as sources for prosocial motivation and commitment are somewhat malleable and improvable through efforts even beyond childhood or adolescence. Previous studies examined how to measure moral implicit theories, which are associated with a belief about malleability and improvability of moral character, and how such incremental theories of morality influenced social attitudes, conceptions, and behaviors, but they did not investigate the developmental outcomes of such theories [7,8]. In fact, developmental psychologists studying prosocial and moral development have demonstrated that one’s moral character can change and be developed even later in one’s life through experiences and external influences. For instance, an exemplar of social justice, Virginia Durr, started to commit herself to social movements after her graduation from college [5]. Virtue moral philosophers have also argued that one’s moral character can be developed and shaped through education, training, and actual prosocial engagement, and is not something fixed and innate [9,10]. Given these studies, it is clear that moral character is improvable throughout the whole life, even if it would be more difficult to change it later in one’s life. Nevertheless, more studies are required to identify the causal relationship between such a belief that moral character can be improved, prosocial motivation, and finally, prosocial behavioral outcomes in the reality.

The present study aimed to examine the causal relationship between a belief regarding the malleability of moral character and motivation to engage in prosocial behavior. We will review the theoretical framework related to such a belief, psychological theories about implicit theories [11]. We will also develop and test the reliability and validity of a measurement for the belief, and conduct studies to examine the causal relationship between the belief and change in prosocial behavior with the measurement.

### Implicit theories of intelligence and ability

With the variation in motivational outcomes of students, much conversation exists about why such differences occur and the many factors that contribute to a student’s success or failures. Particularly, attribution theory focuses on individuals’ interpretations of these outcomes and how they affect motivation, so it might provide us with useful insights about the developmental aspects of motivation [12]. These interpretations of success and failure with the concept of implicit theories suggest that people either have an entity theory or an incremental theory of their intelligence or ability in a certain functional domain [13]. Those with an entity theory have the belief that personal characteristics, such as strengths and weaknesses, are established and people do not have the ability to significantly change. Incremental theorists, however, believe that traits are malleable and the potential for change is possible [7,14]. This view of the potential for change and improvement within a person, termed growth mindset, is accompanied by learning goals and the progress of individuals. Learning goals, as opposed to performance goals focused on outcomes, are centered on the process of gaining knowledge and skills [15]. On the one hand, the possession of learning goals is associated with enhanced motivation to actively cope with challenging situations. On the other hand, the presence of performance goals results in decreased motivation in tasks, such as a tendency to avoid challenging situations and learned helplessness while dealing with extremely difficult situations [16].

As a result, along with attribution theory, motivation can be severely impacted based on a student’s implicit theories. Those with an incremental theory are more likely to focus on effort and what they gain through experience even in challenging situations. These people tend to view failure as a signal to work harder and exert more effort, accepting the challenge. On the other hand, those with an entity theory tend to focus on the result of experience and are more likely to attribute failure to their own personal shortcomings. This makes them more vulnerable to negative affect following a failure and can lead to feelings of helplessness. Furthermore, due to their inclination to perseverate on outcomes, entity theorists are more vulnerable to giving up on an activity if their status is threatened (see Table 1 for summary) [17]. Given these, implicit theories of one’s intelligence or ability significantly influence motivation to engage in activities in various domains associated with such an intelligence or ability.

**Table 1:** Comparing people with two different types of implicit theories.

	Incremental implicit theorists	Entity implicit theorists
Belief about change	Believe that one’s intelligence and ability can change over time	Believe that one’s intelligence and ability are fixed and do not change
Belief about improvement	Believe that it is possible to improve one’s intelligence and ability through efforts	Believe that it is impossible to improve one’s intelligence and ability through efforts
Motivation	Have strong motivation to try hard to master skills	Have no motivation for self-improvement
Goal setting	Set mastery goals	Set performance goals
Reaction and interpretation to a failure	Show a strong will to learn from a failure and perceive it as a signal informing the necessity of more efforts	Show no will to learn from a failure and perceive it as a signal informing lack of one’s ability

### Growth mindset and the promotion of motivation

The presence of a belief that one’s intelligence and ability is malleable and improvable, that is, incremental implicit theories or growth mindset, promotes motivation in various domains including prosocial motivation, which is the topic of the present study. Previous psychological studies have suggested possible pathways between incremental implicit theories and motivation associated with prosocial behavior.

First, in general, possessing the growth mindset enables a person to believe that it is possible to become a better person based on currently available abilities and efforts, promotes self-efficacy, and finally, it results in the generation of motivation [18,19]. As a person has a stronger growth mindset, then the person is more likely to believe that abilities and skills are not innate and are improved through learning processes. Consequently, such beliefs promote self-efficacy in general, and motivation to engage in learning processes [20,21].

A previous review demonstrated that such a pathway between the growth mindset and motivation also exists in the domain of prosociality. In general, a strong self-efficacy was proven as a significant predictor of prosocial behavior in various developmental stages, from childhood to late adolescence [22]. Bandura argued that self-efficacy scores in various domains, including social, self-regulatory, and academic self-efficacy, were positively associated with prosocial behavioral tendency; on the other hand, the scores were negatively correlated with antisocial behavioral tendency, including emotional irascibility, physical and verbal aggression, valuation of aggression, and moral disengagement. Given these results, the presence of growth mindset perhaps promotes self-efficacy in general, and finally, positively influences prosocial motivation and behavioral tendency. A person with the growth mindset in the domain of personality is more likely to confidently believe that it is possible to improve the person’s personality by engaging in prosocial activities, and perhaps has strong prosocial motivation [23]. On the other hand, a person who does not believe that it is difficult to become a better person through efforts is relatively more likely to have the hostile intent attribution bias and aggressive desire, and weaker prosocial motivation compared to the person’s counterpart with the growth mindset [24].

Second, implicit theories of ability can also contribute to the promotion of motivation by helping people set mastery goals, instead of performance goals. A person who has mastery goals in a certain domain strives to master skills pertaining to the domain and underscores the value of effort and growth while mastering such skills [25]. On the other hand, the person’s counterpart with performance goals tries to demonstrate a certain performance, such as a high test score. According to previous studies, a person who possesses incremental implicit theories of intelligence and ability is more likely to acknowledge the person’s effort and growth in intelligence and ability itself as evidence of mastery and to set mastery goals instead of performance goals [26,27]. Finally, setting mastery goals promotes motivation in academic settings [28,29] even under challenging situations [30,31]. Furthermore, the presence of mastery goals is also beneficial for moral and prosocial motivation similar to the case of academic motivation. For instance, setting mastery goals among youth soccer players was positively correlated with motivation to implement sportsmanship behaviors as well as prosocial motivation in general [32]. On the other hand, studies showed that mastery goals were negatively associated with, and performance goals were positively associated with, antisocial behavioral tendency in academic settings, such as tendency to cheat [33,34]. Having mastery goals perhaps makes people value efforts regardless of outcomes, so people are less likely to engage in anti-moral behaviors compared to their counterparts possessing performance goals, because they would value appropriate means and efforts to achieve their goals instead of the goals per se. Thus, the presence of the growth mindset promotes prosocial motivation and behavior in general possibly through the formation of mastery goals.

Hence, incremental implicit theories, the growth mindset, in general contribute to the formation of moral motivation significantly. The promotion of self-efficacy and mastery goals perhaps contributes to such a positive correlation between the growth mindset and formation of prosocial motivation and behavior.

### The current study

The purpose of the present study is to examine whether incremental implicit theories in the domain of morality, the moral growth mindset, promote prosocial behavior by conducting two-wave studies. Although several previous studies examined the relationship between implicit theories of moral character and moral functioning [7,8], as they focused on cognitive aspects of morality and conducted cross-sectional investigations, the causal relationship between such implicit theories and prosocial behavioral outcomes has not been properly studied. Thus, we examine whether the presence of implicit theories of morality influenced changes in service engagement, a form of prosocial behavior, over time through two two-wave studies. Study 1 focused on college students, while Study 2 targeted 8^th^ graders.

Given previous studies that have demonstrated that the presence of the growth mindset promoted motivation for school engagement and social adjustment [11,35,36], we hypothesized that the moral growth mindset would promote service engagement significantly in both age groups. First, given previous studies showing the association between growth mindset and positive motivational outcomes [7,8,24], the main effect of the growth mindset might be significant. Second, as some other studies showed the moderating effect of growth mindset in positive youth development [37–40], the moral growth mindset might also play a significant role as a moderator. Thus, we were interested in whether the main effect or interaction effect of the moral growth mindset influenced service engagement.

## Study 1

We examined the effect of the moral growth mindset on engagement in various service activities. We used the implicit theories of morality survey after testing its reliability and validity. In addition, college students’ service activity engagement was surveyed twice to examine how the moral growth mindset influenced participants’ service engagement.

### Methods

#### Participants

We recruited a total of 127 Korean undergraduate and graduate students (46 males, 80 females, 1 did not specify gender). The average age of participants was 21.14 years (*SD* = 4.12). They were recruited by posting advertisements on social media, including SNUlife.com and Facebook. All of these 127 participants completed our implicit theories of morality survey form. Data collected from these participants was used for the psychometric assessment of our Implicit theories of morality survey. Of these 127 participants, 54 participants (18 males, 36 females) completed both pre- and post-test voluntary service engagement surveys. 73 participants withdrew from our study (attrition rate = 57.48%). The average age of these 54 participants was 22.44 years (*SD* = 5.35). They also reported how many years they had studied in college. They had studied in the university for 3.09 years (*SD* = 1.19) on average.

We performed Little’s missing completely at random (MCAR) test to examine whether the attrition rate was biased in terms of participants’ demographics [41]. Little’s MCAR test has been used in developmental psychological studies to test whether a specific group of participants are more likely to withdraw from a longitudinal investigation. Little’s MCAR test reports whether certain variable(s) cause or correlate with participants’ attrition [42]. The result of Little’s MCAR test [41] indicated that the attrition rate was not significantly different across different genders, χ^2^ (1) distance = .09, *p* = .77.

This study was exempted from IRB review because it was identified as “research involving educational tests, surveys, interviews, or observation of public behavior” by the Stanford University IRB, and “research involving the collection or study of existing data, documents, or records” by the University of Alabama IRB.

### Measures

**Implicit theories of morality survey:** We revised a previously developed measurement for general implicit theories to measure implicit theories in the domain of moral character [26,35,43]. In fact, previous studies that examined the cross-sectional association between implicit theories in the domain of morality and the development of moral judgment used their own measure [7,8]. However, we decided to use our revised measure because the previously invented measure consisted of three items while it has been recommended that a measure includes at least five items to evaluate its psychometrical properties properly [44]. Thus, we decided to revise the previously developed six-item survey form of implicit theories in weight management [43], which is based on Dweck’s six-item implicit measure of intelligence [26]. We replaced terms related to body weight and image in the original measure with terms related to morality and moral character.

The revised measurement included six items. Participants were asked to indicate how much they agree with each statement in the survey form associated with a belief about whether it would be possible to improve their morality and character with efforts. Each item is rated on a seven-point Likert scale anchored at 1 = strongly disagree and 7 = strongly agree. Among the six items, item 1, 2, and 4 are reverse coded items. Items included in the measurement are presented in Table 2.

**Table 2:** Descriptive statistics and reliability indicators (Study 1).

Item	Mean	Median	SD	Skewness	Kurtosis	Item-test correlation (with item 1)	Item-test correlation (without item 1)
1. You have a certain morality and character, and you can’t really do much to improve it. ^a^	4.73	5.00	1.80	-.57	2.21	.47	-
2. Your morality and character are something about you that you can’t improve very much. ^a^	5.37	6.00	1.19	-.86	3.33	.73	.78
3. No matter who you are, you can significantly improve your morality and character.	5.24	5.00	1.20	-.89	3.75	.61	.67
4. To be honest, you can’t really improve your morality and character. ^a^	5.12	5.00	1.32	-.51	2.43	.77	.80
5. You can always substantially improve your morality and character.	4.64	5.00	1.37	-.47	2.43	.83	.86
6. You can improve your basic morality and character considerably.	4.53	5.00	1.27	-.39	2.55	.82	.86

^a^ Reverse coded items.

The reliability of this measurement was estimated by the its internal consistency. The overall calculated Cronbach’s α was .77, which indicates an acceptable reliability [45]. In addition, the calculated Spearman-Brown prophesy reliability estimate was .79 indicating high correlation between even- and odd-numbered items.

The validity was tested by exploratory factor analysis (EFA) and confirmatory factor analysis (CFA). Before performing EFA, we performed assumption tests in order to examine whether EFA was an appropriate method to extract factors from the collected dataset. The calculated determinant of the correlation matrix was greater than 0 (.08) and the Bartlett test of sphericity also reported that the variables are intercorrelated, χ^2^ (15) = 306.78, *p* < .001. The Kaiser-Meyer-Olkin measure of sampling adequacy value was .80. It indicated that EFA could be performed with the dataset. We created a scree plot to determine the number of factors found from the survey. S1 Fig indicates that one factor had an eigenvalue of 1.00 or higher, and the decrease of the eigenvalue became less steep when the number of factors became greater than one. Both the scree plot and the eigenvalue suggested a one-factor model [46]. In addition, analysis of variances explained by factors demonstrated that nearly 100% of variances were explained by one factor and a one-factor model would be appropriate. Table 3 demonstrates the factor loadings of each item. Because we adopted a one-factor model, we did not rotate the resultant matrix.

**Table 3:** Calculated factor loadings from EFA.

	Study 1		Study 2	
	With Item 1	Without Item 1	With Item 1	Without Item 1
Item 1	.19	-	.01	-
Item 2	.70	.70	.62	.62
Item 3	.53	.54	.66	.66
Item 4	.73	.72	.81	.81
Item 5	.85	.85	.84	.84
Item 6	.85	.85	.78	.78

Finally, we conducted CFA to cross-validate the model. S2 Fig demonstrates that all good fit indicators, RMSEA, SRMR, CFI, and TLI, suggested that the current model was appropriate with all six items. However, the average extracted variance (AVE) was .45, smaller than the threshold for acceptable AVE, .50, while the construct reliability (CR) was .81.

**Fig 1:**
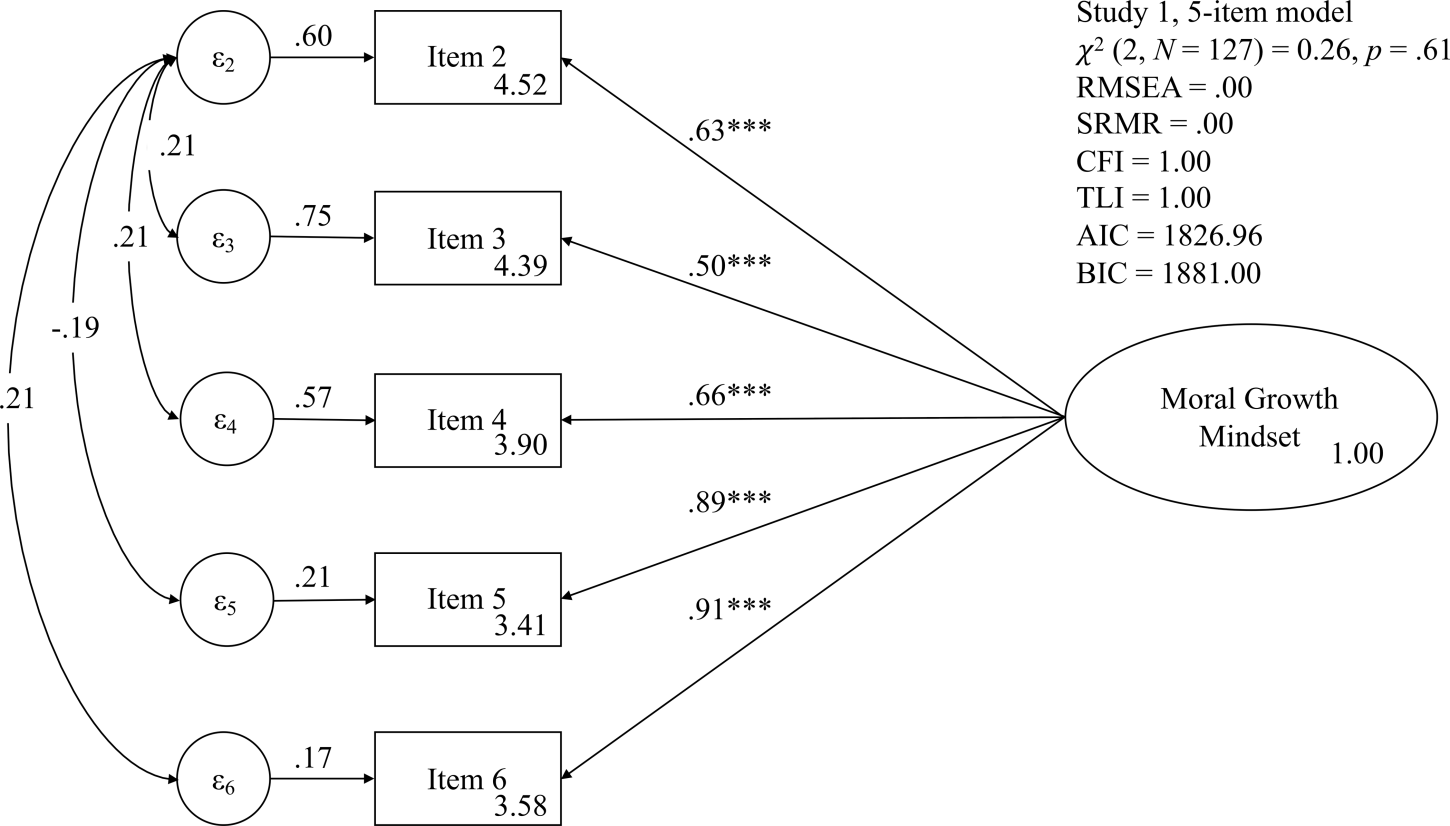
Results of confirmatory factor analysis of the implicit theories of morality survey form without item 1 in Study 1. *** *p* < .001.

Thus, we excluded item 1 showing the smallest factor loading. The Cronbach’s α became .85 and Spearman-Brown prophesy reliability estimate became .85. When we performed EFA again, the calculated determinant of the correlation matrix was greater than 0 (.09) and the Bartlett test of sphericity also reported that the variables are intercorrelated, χ^2^ (10) = 301.76, *p* < .001. The Kaiser-Meyer-Olkin measure of sampling adequacy value was .80. The scree plot (see S3 Fig) and eigenvalue also indicated that a one-factor model was the most adequate. The resultant factor loadings for all items became greater than .5 (see Table 2). Fig 1 shows the good CFA fit indices of this 5-item model. The calculated AVE was .54 and CR was .85 indicating good reliability and validity. Hence, we decided to use the 5-item form without item 1.

#### Voluntary service engagement survey

We distributed a form inquiring actual service engagement to the participants. In order to minimize the possibility of fake reports, we requested them to provide us with concrete information about the name of organizations that they have participated in and the length of engagement (see S1 Text for the form).

#### Procedures

We sent a link to an anonymized Qualtrics survey form to all 127 participants who were initially recruited. They completed the implicit theories of morality survey and demographic survey online. Then, we sent an additional online survey form, the voluntary service engagement survey form, to the participants. Of the 127 participants, fifty-four completed the service engagement survey form. For analysis of change in service engagement, we contacted the participants six weeks after the initial survey session and asked them to complete the voluntary service engagement survey form again. We asked them to report their voluntary service activity experience during the month immediately preceding the time of each survey session.

We analyzed the influence of the moral growth mindset in the change in voluntary service engagement with regression analysis. First, we examined whether initial service engagement explained the post-test service engagement significantly while controlling for demographic variables, i.e., gender, age, and years of college-level education. Second, we added the main effect of the moral growth mindset to the regression model. Third, the interaction effect of the moral growth mindset and initial service engagement was added to the model. We tested whether the added variables improved the model significantly.

### Results

#### Descriptive statistics

The descriptive statistics of variables of interest, i.e., the moral growth mindset and voluntary service engagement, are presented in S1 Table. When we compared initial and post-test service engagement, the difference was insignificant, *t* (106) = -.48, *p* = .63, *d* = .09.

#### Multiple regression analysis

**Fig 2:**
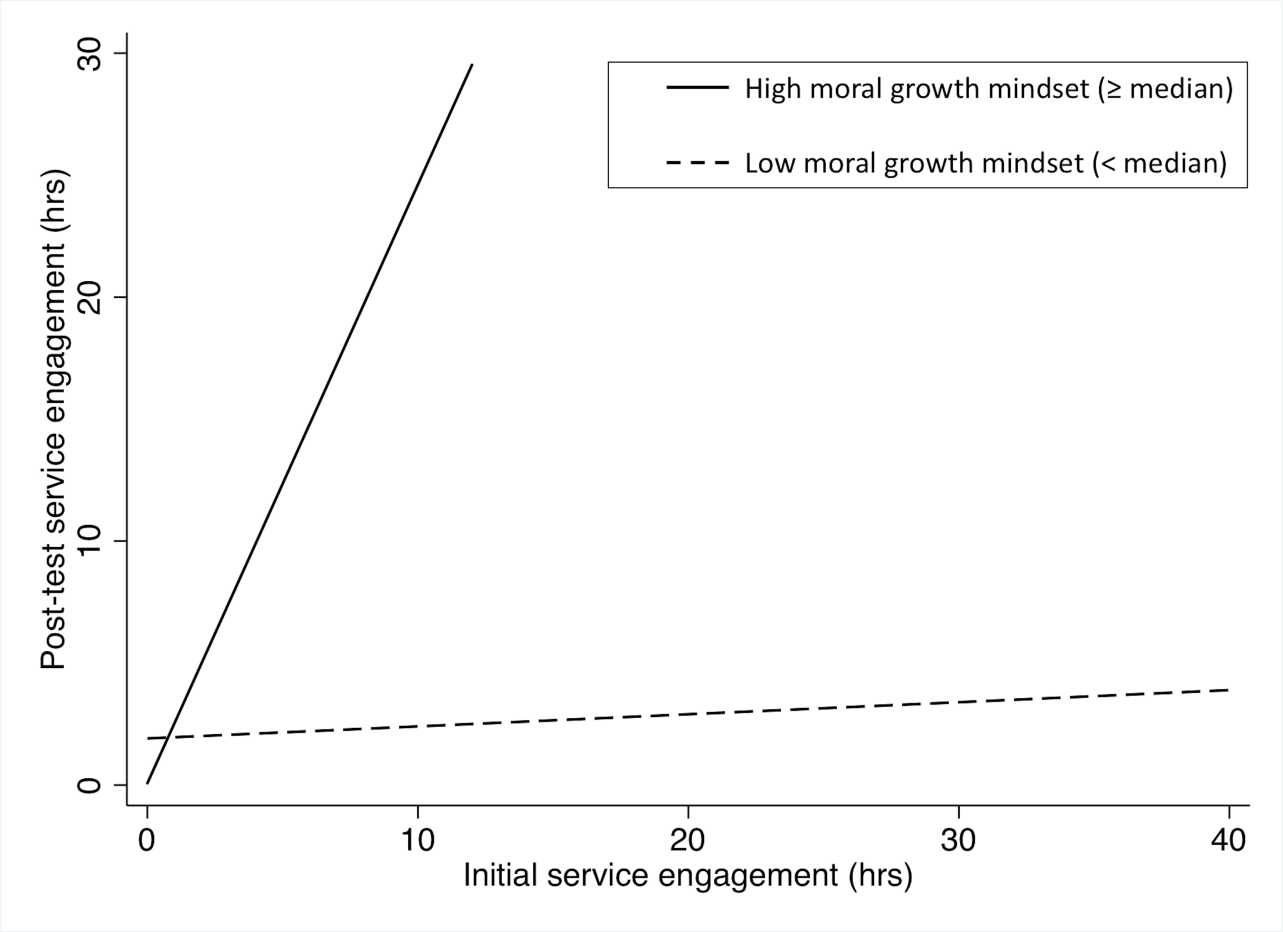
Change in service engagement among high and low moral growth mindset score groups.

The results of regression analyses are presented in Table 4. When we compared three regression models, the third model including the interaction effect of the moral growth mindset and initial service engagement was acceptable given the result of F-test and significance of estimated coefficients. In this model, both the interaction effect between the moral growth mindset and initial engagement, and the main effect of initial engagement were statistically significant. Given the significant interaction effect, we found that the moral growth mindset moderated the relationship between initial and post-test service engagement significantly (see Fig 2). The effect size of the interaction effect of the moral growth mindset by initial engagement, *f* = .44, and that of the main effect of initial engagement, *f* = .41, were medium.

**Table 4:** Estimated regression coefficients in Study 1.

	Model 1	Model 2	Model 3
Controls			
Gender	-1.79	-1.79	-2.73
Age	-.01	-.01	-.02
Years of college education	1.12	1.12	.98
Main effects			
Initial service engagement	.27	.27	-5.19**
Moral growth mindset		.01	-1.40
Interaction			
Initial service engagement x moral growth mindset			1.17**
*F* total	1.52	1.19	3.20
Adjusted *R*^*2*^	.04	.02	.20
Δ*F*		.00	11.88**
Δ*R*^*2*^		.00	.18

Estimated coefficients were standardized.

** *p* < .01.

*** *p* < .001.

### Discussion

Findings from the regression analysis demonstrated that the interaction between initial service engagement and moral growth mindset influenced the post-test service engagement significantly. As the interaction effect was significantly positive, the presence of moral growth mindset is perhaps a protective factor in the promotion of prosocial behavior, which was represented by service engagement in this study.

These findings are in line with the previous developmental psychological studies examining the role of growth mindset in positive youth development. The presence of growth mindset is significantly associated with motivation for self-improvement in various domains, including but not limited to, academic achievement, social adjustment, and bullying prevention behavior [11,14,35,36]. The impact of the growth mindset is also significant for personality change and improvement; more specifically, a belief that personality is malleable and can be developed through efforts and education promotes motivation to have better personality [23]. In addition, the significant interaction found in the present study is coherent with previous studies reporting the significant moderating effect of growth mindset on the relationship between stressful life and mental health [37], parenting and subjective well-being [38], and longitudinal change in social anxiety [39].

Thus, it is perhaps the case that having a belief that it is possible to become a morally better person through moral engagement, that is, the moral growth mindset, promotes prosocial engagement significantly. Coherently, we demonstrate that participation in prosocial service activity was positively moderated by the moral growth mindset in this study.

## Study 2

We examined the effect of the moral growth mindset on the change in service engagement among Korean 8^th^ graders. We conducted this study in order to examine whether such an effect of the moral growth mindset was also significantly among a different age group.

### Methods

#### Participants

187 Korean 8^th^ graders (98 males, 89 females) in a middle school located in Seoul Metropolitan area, Korea, participated in this study. All participants were fourteen years old at the time of data collection. They completed the implicit theories of morality survey form. As we did in Study 1, we collected pre- and post-test voluntary service engagement data in addition to the implicit theories of morality data. These 187 participants also completed the pre-test service engagement survey form. Of the 187 participants, 180 (92 males, 88 females) completed both pre- and post-test service engagement survey forms. 7 participants withdrew from our study (attrition rate = 3.74%).

As we did in Study 1, we performed Little’s MCAR test to examine whether the attrition rate was significantly influenced by or correlated with the surveyed demographics, gender. The result of Little’s MCAR test indicated that the attrition rate was not significantly different across different genders, χ^2^ (1) distance = 1.93, *p* = .16. This result indicates that study withdrawal happened randomly and did not occur more frequently in a specific gender group.

This study was exempted from IRB review because it was identified as “research conducted in established or commonly accepted educational settings involving normal educational practices” by the Seoul National University IRB, and “research involving the collection or study of existing data, documents, or records” by the University of Alabama IRB. In addition, all data collection procedures have been reviewed and approved by Stanford University IRB (Protocol ID: 29547). The IRB approved a waiver of parental consent.

### Measures

**Implicit theories of morality survey:** We used our implicit theories of morality survey. We tested reliability and validity of the measurement before applying it to 8^th^ graders. The calculated Cronbach’s α was .73 (see Table 5 for descriptive statistics and additional reliability indicators). The Spearman-Brown reliability estimate, .81, also indicated good reliability.

**Table 5:** Descriptive statistics and reliability indicators (Study 2).

Item	Mean	Median	SD	Skewness	Kurtosis	Item-test correlation (with item 1)	Item-test correlation (without item 1)
1. You have a certain morality and character, and you can’t really do much to improve it. ^a^	5.21	6.00	1.33	-.60	2.77	.24	-
2. Your morality and character are something about you that you can’t improve very much. ^a^	5.12	6.00	1.41	-.44	2.19	.73	.73
3. No matter who you are, you can significantly improve your morality and character.	5.21	6.00	1.41	-.65	2.86	.73	.76
4. To be honest, you can’t really improve your morality and character. ^a^	5.23	6.00	1.37	-.61	2.30	.84	.85
5. You can always substantially improve your morality and character.	4.96	5.00	1.42	-.29	2.55	.84	.86
6. You can improve your basic morality and character considerably.	4.64	5.00	1.26	-.09	2.53	.78	.82

^a^ Reverse coded items.

The results of assumption tests indicated that EFA was performable with the collected data. The determinant of the correlation matrix was .08, and the result of Bartlett test indicated that variables were intercorrelated, χ^2^ (15) = 455.10, *p* < .001. The calculated Kaiser-Meyer-Olkin Measure, .80, was meritorious. EFA reported that all items loaded together on a single factor given the eigenvalue, amount of explained variances by factors, and scree plot (see S4 Fig). However, the factor loading of item 1 was very small, .00 (see Table 3).

Second, CFA reported that the model with all 6 items demonstrated good fit indicators (see S5 Fig). However, the path from the moral growth mindset to item 1 was statistically insignificant, *p* = .66 while the paths from the moral growth mindset to other items were statistically significant, *p* < .001. In addition, the calculated AVE, .47, and CR, .82, showed the inadequate AVE.

**Fig 3:**
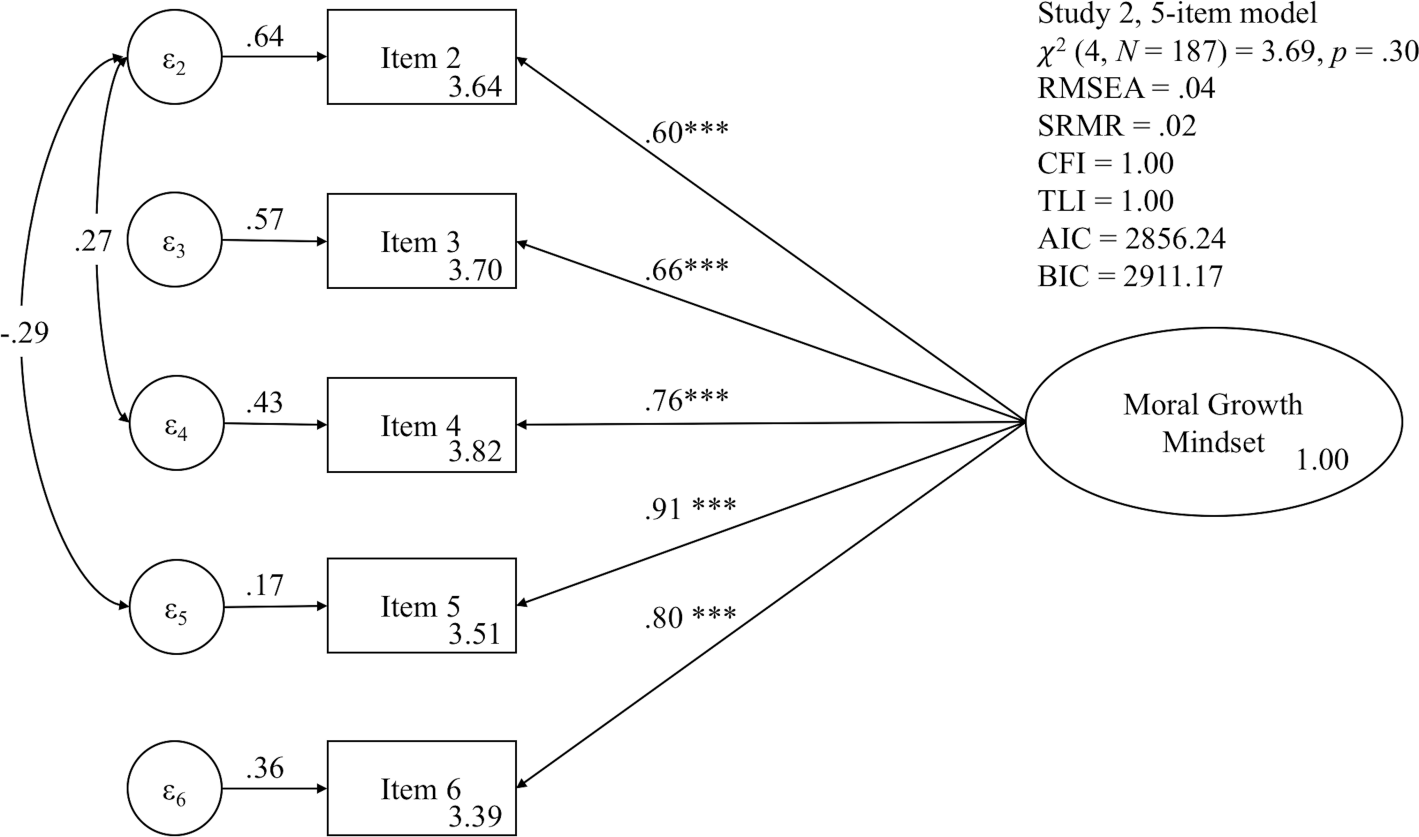
Results of confirmatory factor analysis of the implicit theories of morality survey form without item 1 in Study 1. *** *p* < .001.

Thus, we decided to exclude item 1 from the survey form. We recalculated reliability indicators, Cronbach’s α and Spearman-Brown reliability estimate, after excluding item 1 as recommended by validity indicators. All reliability indicators suggested that the exclusion of item 1 was able to improve the overall reliability of the survey. Cronbach’s α increased from .73 to .86. In addition, the Spearman-Brown reliability estimate also increased from .80 to .84. In the case of EFA, the recalculated determinant of the correlation matrix was .09, and the result of Bartlett test indicated that variables were intercorrelated, χ^2^ (10) = 445.81, *p* < .001. The recalculated Kaiser-Meyer-Olkin Measure, .82, was meritorious. Given the scree plot and eigenvalue, we assumed a one-factor model (see S6 Fig). All factor loadings were greater than .6. Finally, CFA fit indicators were good (see Fig 3). Both the AVE, .57, and CR, .87, became acceptable. Hence, we decided to use this 5-item version for this study as we did in Study 1.

**Youth service engagement survey:** We measured participants’ voluntary service activity engagement during the last two months with a questionnaire previously used in civic and community service purpose studies [2,3,47,48]. The survey form was designed to measure the frequency of engagement in service activity offered by: 1. Religion, 2. Charity in general that does not solely focus on youths, 3. Art, and 4. Child-adolescent-student community organizations. Each item is rated on a five-point Likert scale (1 = none, 2 = once or twice, 3 = sometimes, 4 = almost every week, and 5 = more than once per week).

#### Procedures

We contacted a teacher in the middle school where all participants were enrolled for the survey. The teacher distributed survey forms to participating students in classrooms. The participants were asked to complete all survey forms during a class hour (45 minutes). Meanwhile, the teacher stayed in a separated area in the classrooms to allow the participants to answer survey questions autonomously. At the end of the class hour, the teacher collected completed survey forms from the participants. The post-test survey session was conducted twelve weeks after the initial survey session. We asked the participants to report their service engagement during the two months immediately preceding the time of each survey session.

Similar to Study 1, we examined the effect of the moral growth mindset on the change in service engagement. We conducted multiple regression analysis to examine such an effect. Three different regression models were evaluated: first, only with the main effect of initial engagement; second, with the main effect of the moral growth mindset; third, with the interaction effect of the moral growth mindset by initial engagement. We included a demographic variable, gender, to control for any gender effect. Participants’ age and years of education were not included in the models, because only fourteen-year old 8^th^ graders were recruited.

### Results

#### Descriptive statistics

Descriptive statistics regarding the moral growth mindset and participation in the four different types of service activity are presented in S2 Table. We compared initial and post-test engagement by conducting t-tests. The decline in service engagement was significant in the case of engagement in art-related organization, and was marginal in the case of engagement in religious organization. Engagement in general charities or youth-related organizations did not change significantly.

#### Multiple regression analysis

**Fig 4:**
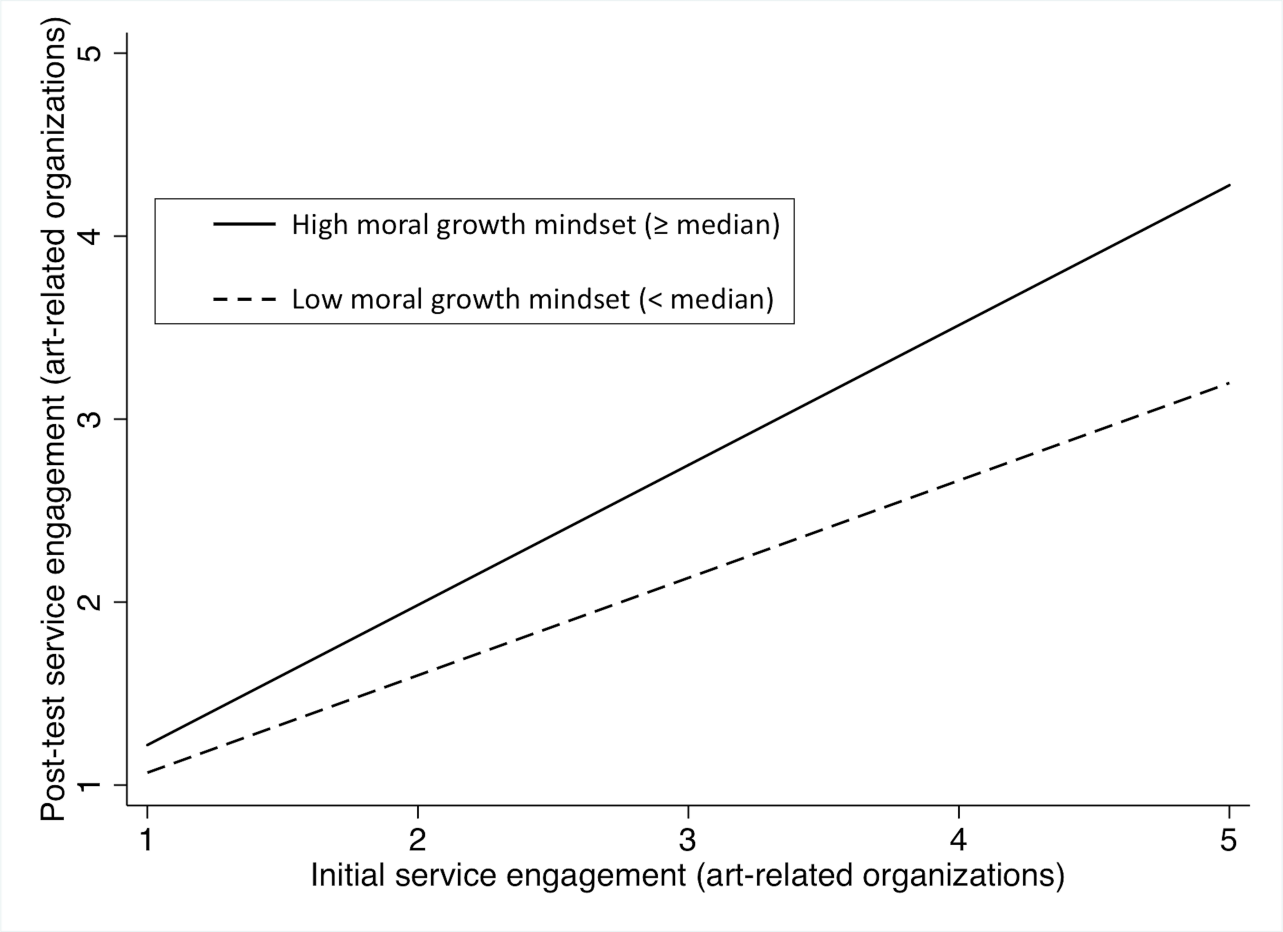
Change in engagement in activities offered by art-related organizations among high and low moral growth mindset score groups.

**Fig 5:**
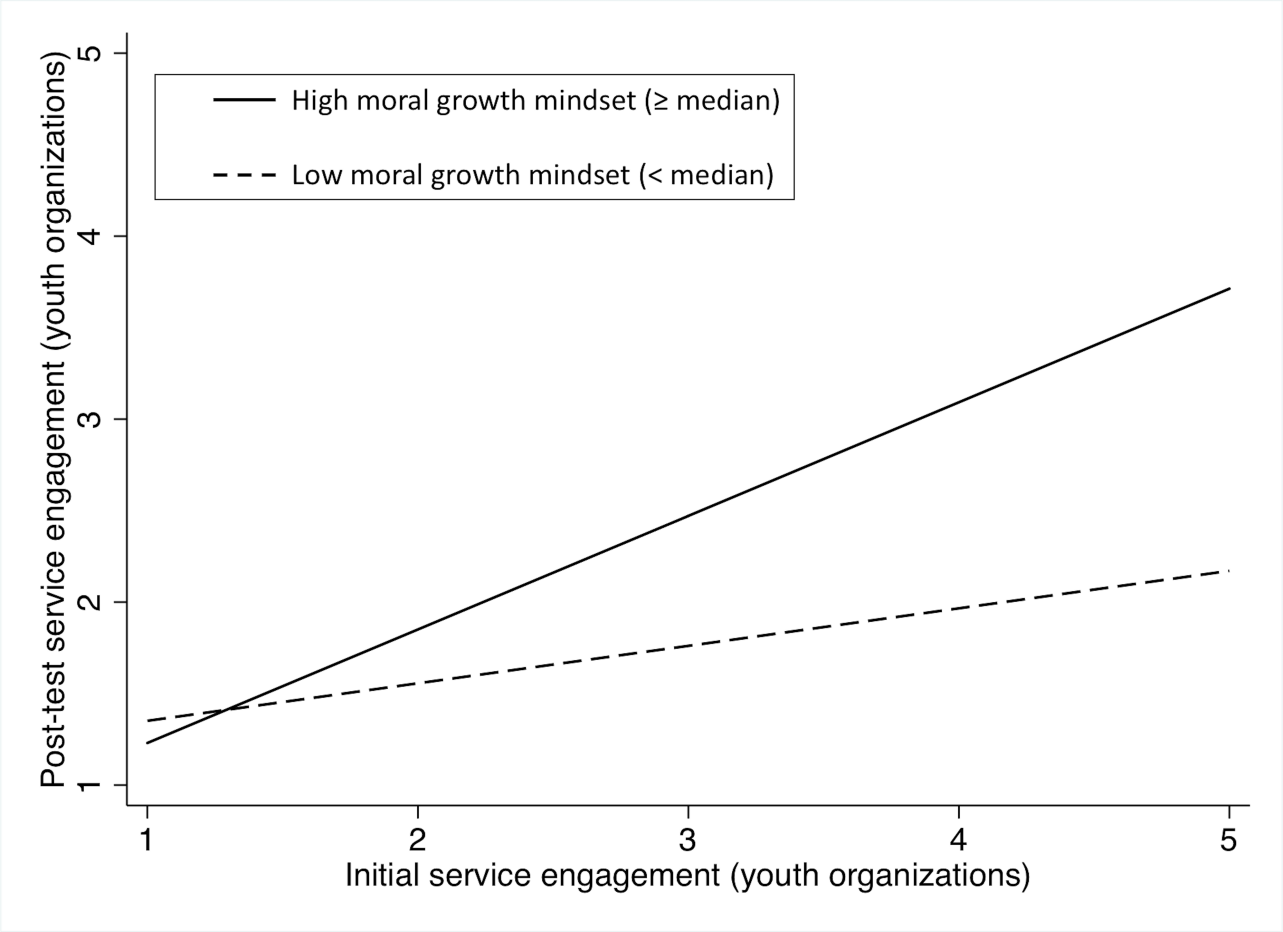
Change in engagement in activities offered by youth-related organizations among high and low moral growth mindset score groups.

We conducted multiple regression analysis with three different models for four dependent variables, i.e., the post-test engagement in four different service activity domains (see Table 6 for results). In the cases of engagement in religious organizations or general charities, only the main effect of initial engagement demonstrated statistical significance (see Fig 4); the moral growth mindset did not influence the post-test engagement significantly in these domains. In the case of engagement in art-related organizations, model 2 showed the highest adjusted *R*^*2*^. In this domain, the main effects of the moral growth mindset as well as that of initial engagement were significant (model 2); the effect size of the moral growth mindset was small, *f* = .13, while that of initial engagement was medium, *f* = .47. When we analyzed participation in youth-related organizations, model 3 showed the best adjusted *R*^*2*^. In this model, the interaction effect of the moral growth mindset by initial engagement was significant (model 3; see Fig 5); its effect size was small, *f* = .17. However, the main effect of the moral growth mindset was insignificant.

**Table 6:** Estimated regression coefficients in Study 2.

	T2 Engagement in Religion			T2 Engagement in Charity		
	Model 1	Model 2	Model 3	Model 1	Model 2	Model 3
Controls						
Gender	.06	.05	.06	-.02	-.03	-.03
Main effects						
Initial engagement	.55***	.55***	.28	.45***	.44***	.53†
Moral growth mindset		.03	-.06		.06	.10
Interaction						
Initial engagement x moral growth mindset			.29			-.10
*F* total	34.85	23.20	17.64	20.42	13.85	10.35
Adjusted *R*^*2*^	.30	.29	.29	.19	.19	.18
Δ*F*		.23	.97		.76	.09
Δ*R*^*2*^		.00	.00		.00	.00
	T2 Engagement in Art			T2 Engagement in Youth		
	Model 1	Model 2	Model 3	Model 1	Model 2	Model 3
Controls						
Gender	.00	-.02	.00	.10	.10	.11
Main effects						
Initial engagement	.46***	.44***	-.02	.49***	.49***	-.41
Moral growth mindset		.15*	-.09		-.10	-.31*
Interaction						
Initial engagement x moral growth mindset			.55			1.04**
*F* total	21.23	15.90	12.28	27.02	17.97	15.93
Adjusted *R*^*2*^	.20	.22	.22	.23	.23	.26
Δ*F*		4.37*	1.30		.14	7.69*
Δ*R*^*2*^		.02	.01		.00	.03

† *p* < .1.

* *p* < .05.

** *p* < .01.

*** *p* < .00

### Discussion

We found that the presence of moral growth mindset influenced students’ engagement in various service activities, particularly activities offered by art-related and youth-related organizations. The moral growth mindset contributed directly to post-test engagement in art-related organizations. In the case of participation in youth-related organizations, the moral growth mindset moderated the relationship between initial and post-test engagement. These findings are in line with Study 1 and previous studies that demonstrated the presence of growth mindset positively influenced motivation in various domains of personality and social behavior [14,23,35,36].

Interestingly, such an impact was not found in the case of engagement in religious organizations or general charities. We might consider the nature of each type of service activity. First, the attainability and relevance of each activity perceived by students perhaps influenced their motivation. Previous social psychological experiments have demonstrated that when students were presented with role models, students were more strongly motivated as the presented models were perceived to be more attainable and relevant to them. More specifically, students were more likely to emulate the presented role models when the achievements of the models were emulatable with a reasonable amount of efforts or the models shared the same interests and backgrounds [49–51]. Second, the availability of and accessibility to each type of service activity might also be significant. Previous psychological experiments have shown that a mere advertisement and persuasion might not induce actual behavioral outcomes; instead, concrete accessible behavioral options and plans should be provided in order to promote behavioral motivation effectively [51–55]. Given these, because activity opportunities offered by art-related and youth-related organizations might be more accessible and relevant to the students, the motivating effect of the moral growth mindset was perhaps significant in these activity domains. On the other hand, activities offered by religious organizations or general charities could be attainable and relevant to students who had a religion or interest in community service in general.

However, the effect of the moral growth mindset on service engagement in two domains was significant, but the findings were mixed. In the case of participation in art-related organizations, the main effect of the moral growth mindset was significant. Whereas the interaction effect between the moral growth mindset and initial engagement was found significant and positive in the domain of youth-related activity, the main effect of the moral growth mindset was negative in this case. In fact, some previous studies examining the psychological impact of the growth mindset have reported the significant main effect of the growth mindset, while others have reported the significant interaction effect between the growth mindset and other psychological or social factors [37–39,56]. However, it is still unclear in which context and condition the main effect or interaction effect significantly influences psychological outcomes. Thus, future studies with a more sophisticated design should be conducted to illuminate why the effect of this factor is differentiated across different domains of service activity.

## General discussion

Our study demonstrated the influences of the moral growth mindset on engagement in various service and civic activities as proxies for motivation to engage in prosocial behavior. We found significant contribution of the moral growth mindset on the change in service engagement in various domains. These findings are in line with previous developmental psychological studies that have reported the positive influences of the growth mindset on the development of intelligence [11,57] and social adjustment [14,23,35,36].

The overall findings from our study suggest that believing that moral character is malleable and improvable through efforts can positively influence motivation to engage in prosocial behavior. The presence of such a growth mindset can encourage people to initiate prosocial behavior by believing that such prosocial behavior is valuable and helps them become a morally better person eventually. Given the previous developmental psychological studies that have demonstrated the impact of implicit theories on motivational processes [23,27], it is plausible that the incremental mindset in the domain of morality also promotes prosocial motivation. On the other hand, if a person possesses a fixed mindset in the domain of morality, which means that morality is somewhat fixed, innate, and cannot be modified or improved through activities, such a mindset is unlikely to promote moral development (e.g., increase in prosocial motivation), because it can weaken the person’s belief that it is possible to become a morally better person through efforts. Thus, the presence of incremental theory of morality, the moral growth mindset, is able to constitute the foundation for the development of prosocial motivation in the long term.

Interestingly, such an impact of the moral growth mindset was limited in the cases of service activities offered organizations that were more likely to be available and relevant to participants. The results of Study 2 showed that the impact of the moral growth mindset existed only in the cases of engagement in art-related or youth-related service activities. Previous studies in fact examined the impact of the growth mindset on academic and social abilities that are closely connected to students’ lives, e.g., academic achievement [11], strategies to deal with bullying [36,58]. As the issues associated with these domains might seem to be relevant and important to the students, the implicit theories in these domains might significantly influence students’ motivation. On the other hand, in the case of engagement in activities offered by religious organizations or general charities, the impact of the moral growth mindset was insignificant. Only students who have a religion or are strongly interested in general charities and social welfare in general might pay their attention to and actively engage in such organizations. As a result, the impact of moral growth mindset would be limited to certain domains of service activities that were relevant and accessible to students. Given these, incremental implicit theories perhaps promote motivation only in the domain in which one is interested in and connected to.

In addition to the aforementioned factors related to the perceived attainability and relevance of specific activity domains, let us consider contextual factors among Korean middle schoolers that might differentiate the influence of students’ moral growth mindset on participation in different activity domains. First, Korean middle schoolers have more opportunities to participate in activities offered by art-related and youth-related organization compared with those offered by other types of organizations. According to a national-wide survey conducted by a Korean governmental institute, 96.1% of Korean adolescents participated in youth-related activities and 85.2% of Korean adolescents participated in art-related activities [59]. However, relatively less Korean middle schoolers are likely to engage in activities offered by religious organization or general charities. Another governmental survey found that only 49.4% of Korean adolescents were affiliated with religion and 45.1% of Korean adolescents voluntarily participated in service activities [60,61]. Given these survey results, accessibility to activities offered by art-related and youth-related organizations is relatively high in Korea, so it might result in the stronger promotional effect of moral growth mindset on activity engagement in those domains. Second, students’ personal interest and school requirements might also contribute to the differentiated effect of moral growth mindset. According to a previous qualitative study examining school-level policies about service activities and students’ service engagement intention, Korean secondary schools are required to employ various service activities in their curriculum [62]. Moreover, this study also reported that many students and parents were concerned about fulfilling service activity requirements set by schools [62]. Hence, students are perhaps likely to pay attention to activities provided or required by their schools, art- and youth community-related activities in particular [59], so behavioral changes in service activities are also likely to occur in the aforementioned activity domains.

However, several limitations should be addressed by conducting future studies. First, although we calculated one score, moral growth mindset, from responses to our revised measurement containing both incremental and entity theories items [26,43], some previous studies calculated two separate scores for incremental and entity implicit theories [63,64]. In fact, denying the possibility to improve morality (low incremental implicit theories) does not necessarily imply that believing that morality is fixed (high entity implicit theories). Thus, future research may need to examine whether those two theories in the domain of morality should also be assessed and interpreted separately by employing a different measurement and scoring method. Second, we focused on a specific domain of human morality, engagement in service activity, in the present study. Whether the moral growth mindset also influences other types of moral functioning and behavior, e.g., moral judgment [65], moral sensibility [66], donating behavior [67], is still unclear and should be examined in future research. Third, we used self-report measures to measure participants’ engagement in service activity. Given the possibility of a social desirability bias [68], the utilization of such self-report measures while examining prosocial behavior would produce unreliable results. Although we used a more structuralized reporting form inquiring concrete information regarding service engagement, e.g., participation periods and lengths, in Study 1 to minimize such bias, it would be a critical issue in Study 2 that used general self-report measures. Future studies may have to use research methods that are less susceptible to social desirability bias, e.g., behavioral observation, neuroimaging methods [69], to address this issue. Fourth, we conducted all studies in Korean schools. Given previous studies reporting differences in moral functioning between Eastern and Western cultures [70–72] and cross-national differences in educational programs dealing with moral and character education [73], the association between the moral growth mindset and prosocial behavior may differ in other cultural contexts. Thus, more studies should be conducted in other countries for a better generalization. Fifth, although we examined the influences of the moral growth mindset, we could not investigate more long-term influences by collecting multi-wave data. More long-term, multi-wave longitudinal studies using the moral growth mindset survey should be conducted to understand how this psychological construct influences the development of prosocial motivation in the life-span.

## Concluding remarks

In the present study, we showed that the moral growth mindset positively influenced motivation to engage in service activities. As previous developmental psychological studies showed the importance of the growth mindset in motivation in general, prosocial motivation is also positively affected by the moral growth mindset as well. Moral educators may consider implementing interventions targeting moral implicit theories on top of traditional moral educational programs focusing on moral development in order to improve the effectiveness of moral education by making students believe that their moral character can be developed by actively participating in moral educational activities.

## Supporting information

S1 Fig Scree plot of eigenvalues in Study 1 (6-item model). (PDF)

S2 Fig Results of confirmatory factor analysis of the implicit theories of morality survey form with item 1 in Study 1. *** *p* < .001.
(PDF)

S3 Fig Scree plot of eigenvalues in Study 1 (5-item model). (PDF)

S4 Fig Scree plot of eigenvalues in Study 2 (6-item model). (PDF)

S5 Fig Results of confirmatory factor analysis of the implicit theories of morality survey form in Study 2 (6-item model). *** *p* < .001.
(PDF)

S6 Fig Scree plot of eigenvalues in Study 2 (5-item model). (PDF)

S1 Table Initial and post-test descriptive and t-statistics in Study 1. (PDF)

S2 Table Initial and post-test descriptive and t-statistics in Study 2. (PDF)

S1 Text Voluntary service engagement reporting form. (PDF)
