# Supplementary figures and images for: Moral growth mindset is associated with change in voluntary service engagement
Source: PLoS One. 2018 Aug 15;13(8):e0202327. doi: 10.1371/journal.pone.0202327 (PMC6093698; doi:10.1371/journal.pone.0202327)

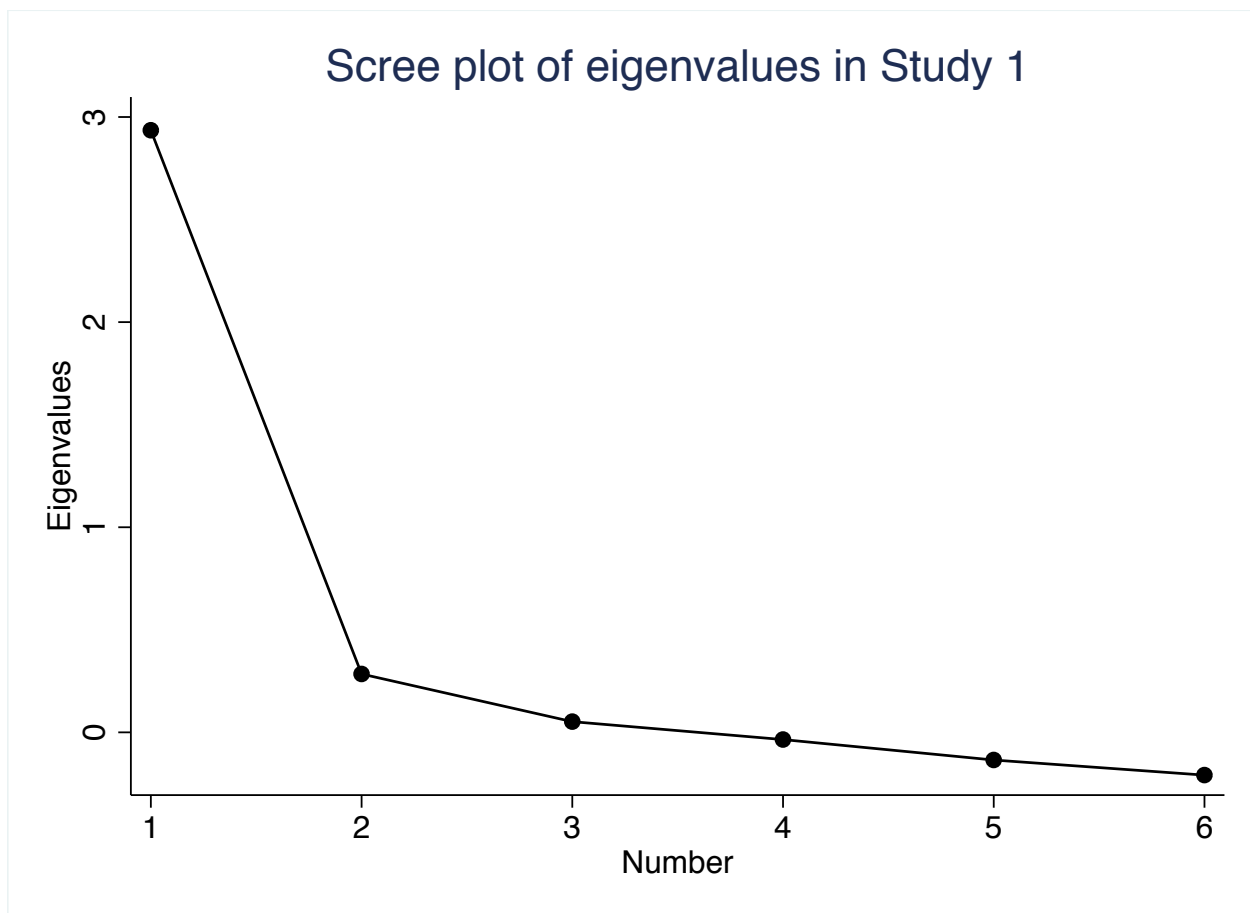

*S1 Fig.* Scree plot of eigenvalues in Study 1 (6-item model).

Supplement: S1 Fig — (PDF) [file pone.0202327.s001.pdf]

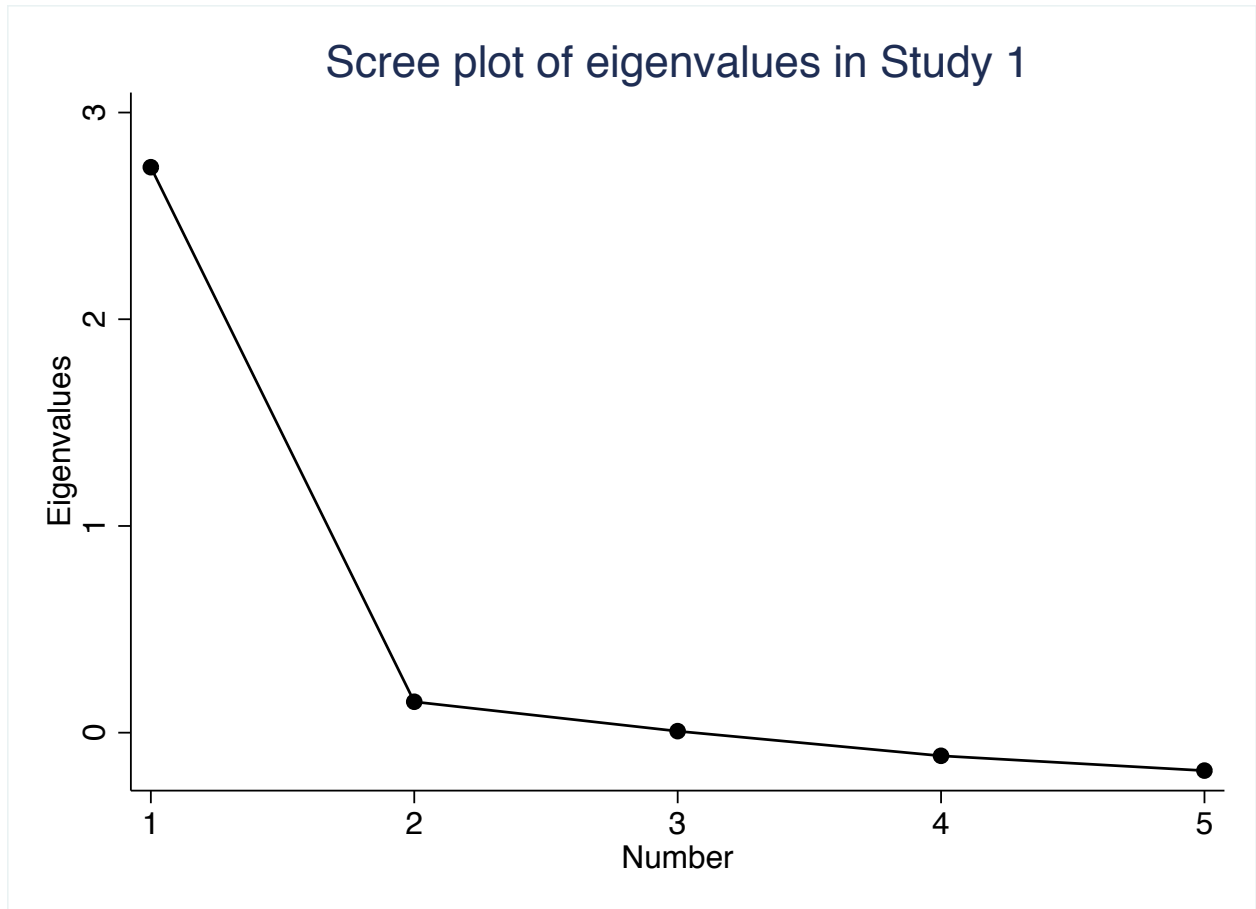

*S3 Fig.* Scree plot of eigenvalues in Study 1 (5-item model).

Supplement: S3 Fig — (PDF) [file pone.0202327.s003.pdf]

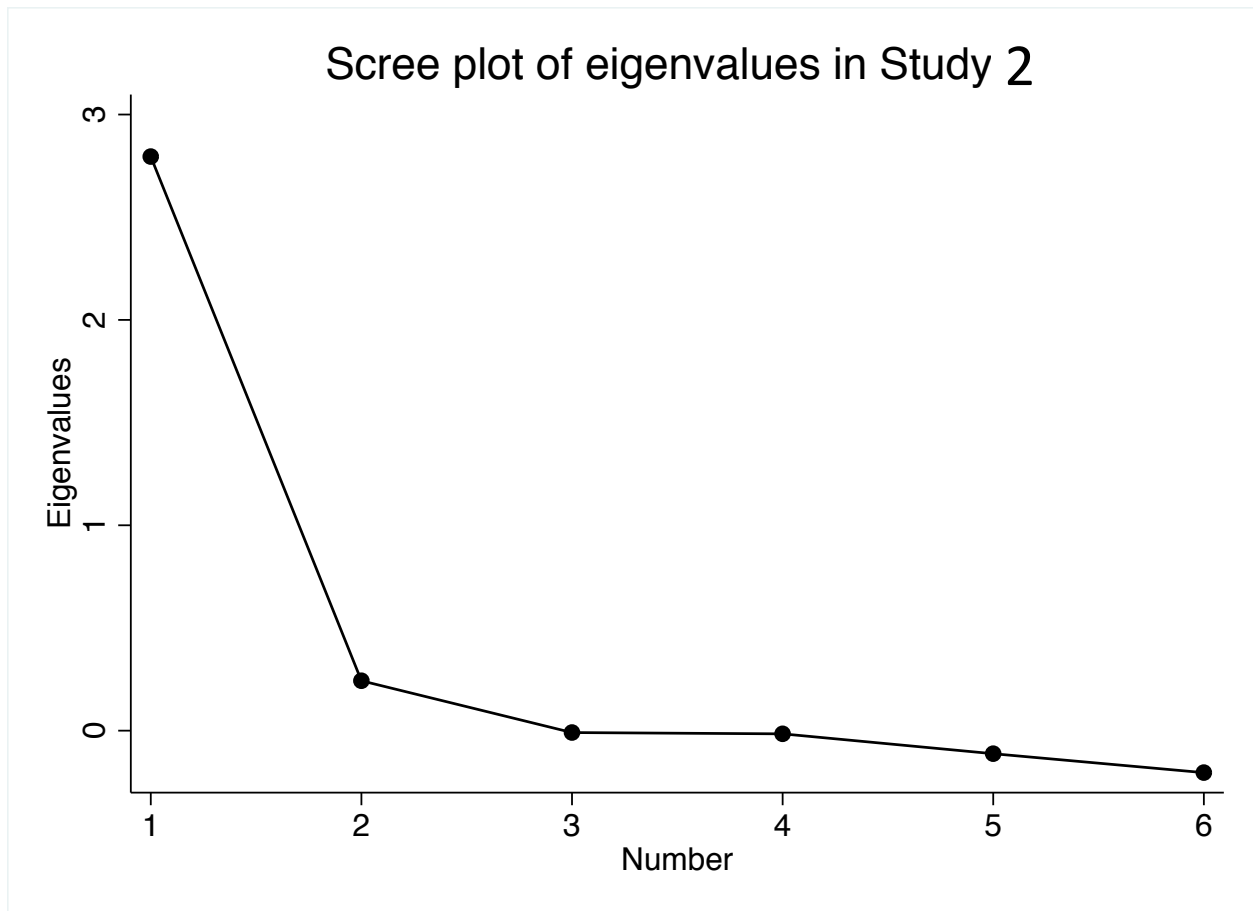

*S4 Fig.* Scree plot of eigenvalues in Study 2 (6-item model).

Supplement: S4 Fig — (PDF) [file pone.0202327.s004.pdf]

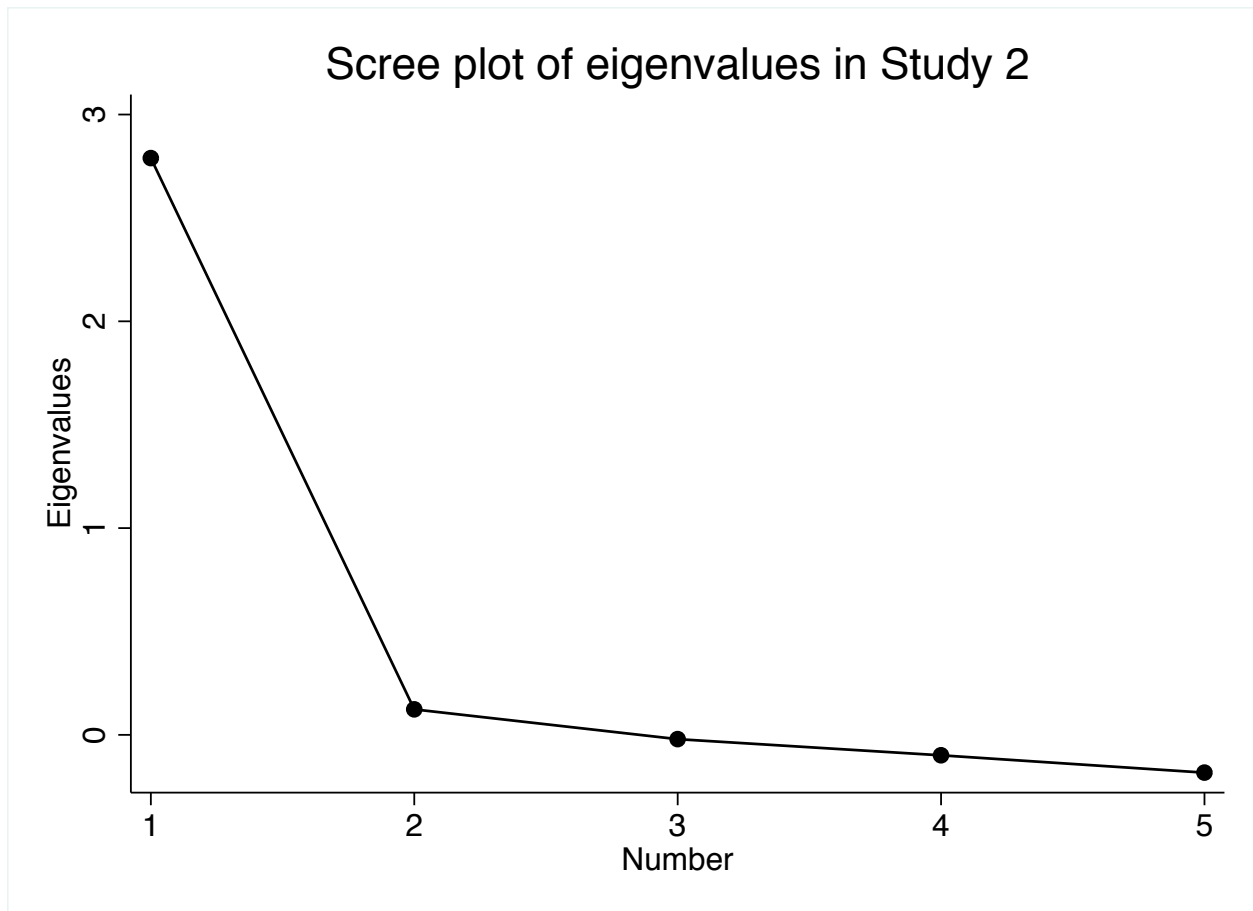

S6 Fig. Scree plot of eigenvalues in Study 2 (5-item model).

Supplement: S6 Fig — (PDF) [file pone.0202327.s006.pdf]
