# Supplementary material for: Moral growth mindset is associated with change in voluntary service engagement
Source: PLoS One. 2018 Aug 15;13(8):e0202327. doi: 10.1371/journal.pone.0202327 (PMC6093698; doi:10.1371/journal.pone.0202327)
Supplement: S5 Fig — *** p < .001. (PDF) [file pone.0202327.s005.pdf]

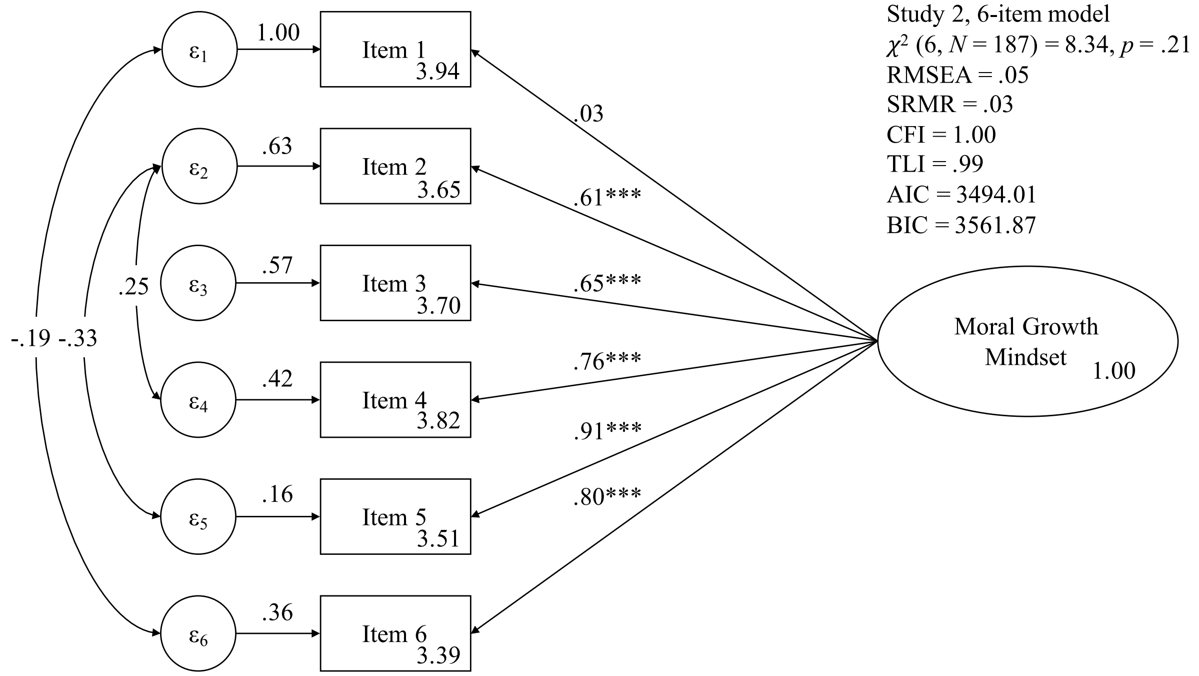

S5 Fig. Results of confirmatory factor analysis of the implicit theories of morality survey form in Study 2 (6-item model). *** $p < .001$.
