# Supplementary material for: Moral growth mindset is associated with change in voluntary service engagement
Source: PLoS One. 2018 Aug 15;13(8):e0202327. doi: 10.1371/journal.pone.0202327 (PMC6093698; doi:10.1371/journal.pone.0202327)
Supplement: S1 Table — (PDF) [file pone.0202327.s007.pdf]

S1 Table

Initial and post-test descriptive and t-statistics in Study 1

Variable	Initial survey ($N = 54$)		Post-test survey ($N = 54$)				
	M	*SD*	*M*	*SD*	*t*	*p*	*d*
Moral growth mindset	4.92	.98	-	-	-	-	-
Voluntary service engagement	1.99	6.11	2.63	7.58	-.48	.63	-.09
