# Supplementary material for: Moral growth mindset is associated with change in voluntary service engagement
Source: PLoS One. 2018 Aug 15;13(8):e0202327. doi: 10.1371/journal.pone.0202327 (PMC6093698; doi:10.1371/journal.pone.0202327)
Supplement: S2 Table — (PDF) [file pone.0202327.s008.pdf]

S2 Table

Initial and post-test descriptive and t-statistics in Study 2

Variable	Initial survey (N = 183)		Post-test survey (N = 183)				
	M	SD	M	SD	t	p	d
Moral growth mindset	4.63	1.12	-	-	-	-	-
Service engagement							
1. Religion	1.38	.89	1.52	1.02	-1.87	.06†	-.29
2. Charity	1.50	.80	1.50	.84	-.09	.93	-.01
3. Art	1.10	.40	1.21	.65	-2.51	.01*	-.39
4. Child-adolescent-student community	1.67	.85	1.61	.88	1.02	.31	.15

† $p < .1$. * $p < .05$.
