# Supplementary material for: Moral growth mindset is associated with change in voluntary service engagement
Source: PLoS One. 2018 Aug 15;13(8):e0202327. doi: 10.1371/journal.pone.0202327 (PMC6093698; doi:10.1371/journal.pone.0202327)
Supplement: S1 Text — (PDF) [file pone.0202327.s009.pdf]

S1 Text

Voluntary Service Engagement Reporting Form

Please write your experience of voluntary services during the last month (from . . . to . . .).

| Period (If you are not sure of the exact date, please provide the date as you remember) | Name of the charity | Amount of time (hours) |
|---|---|---|
| Ex) May. 5. 2013. | Save the Children | 3 |
| | | |
| | | |
| | | |
| | | |
| | | |
| (The rest has been omitted) | | |
